# Bark and wood tissues of American elm exhibit distinct responses to Dutch elm disease

**DOI:** 10.1038/s41598-017-07779-4

**Published:** 2017-08-02

**Authors:** S. M. Sherif, L. A. Erland, M. R. Shukla, P. K Saxena

**Affiliations:** 1 Gosling Research Institute for Plant Preservation, Department of Plant Agriculture, University of Guelph, Guelph, ON Canada; 2 Virginia Tech, Alson H. Smith, Jr. Agricultural Research and Extension Center, Winchester, VA USA

## Abstract

Tolerance to Dutch elm disease (DED) has been linked to the rapid and/or high induction of disease-responsive genes after infection with the fungus *Ophiostoma novo*-*ulmi*. Although the fungal infection by *O*. *novo-ulmi* primarily takes places in xylem vessels, it is still unclear how xylem contributes to the defense against DED. Taking advantage of the easy separation of wood and bark tissues in young American elm saplings, here we show that most disease-responsive genes exhibited higher expression in wood compared to bark tissues after fungal infection. On the other hand, the stress-related phytohormones were generally more abundant in the bark compared to wood tissues. However, only endogenous levels of jasmonates (JAs), but not salicylic acid (SA) and abscisic acid (ABA) increased in the inoculated tissues. This, along with the upregulation of JA-biosynthesis genes in inoculated bark and core tissues further suggest that phloem and xylem might contribute to the de novo biosynthesis of JA after fungal infection. The comparison between two tolerant elm varieties, ‘Valley Forge’ and ‘Princeton,’ also indicated that tolerance against DED might be mediated by different mechanisms in the xylem. The present study sheds some light on the amplitude and kinetics of defense responses produced in the xylem and phloem in response to DED.

## Introduction

Dutch elm disease is a vascular wilt fungal disease that led to the decimation of most forest and urban elm populations in North America and Europe, and has been considered the most devastating shade tree disease in the United Sates. Three fungal species from the genus *Ophiostoma* are linked to DED. *O*. *novo-ulmi* is the most aggressive among them and the causal agent of the ongoing pandemic that started in the early 1970’s^1^, which has led to the death of millions of American elm trees in Canada and the US^2^. DED is transmitted by elm bark beetles from the genera *Scolytus* and *Hylurgopinus* ^3–5^. Beetles carry fungal spores on the surface of their bodies and in the gut^5^; and as they feed on and tunnel in twig crotches, they transfer fungal spores to xylem tissues. The proliferation of the fungus inside xylem vessels and the accumulation of tyloses and gels around fungal structures plug xylem vessels and render them non-functional as water-conducting elements. As a result, the infected plants wilt and eventually die. Traditional management approaches for DED, though largely ineffective, include sanitation and the use of registered pesticides and fungicides; or even the use of bio-control vaccines^6^ in order to induce natural defense against DED. Therefore, using the genetic resistance of host plants has been regarded as the most effective approach. Although no resistance against DED has been identified in American elm (*Ulmus americana*), trees which survived the last pandemics are theoretically considered tolerant to DED. Moreover, breeding programs have released American elm cultivars such as ‘Valley Forge,’ ‘Princeton,’ ‘Delaware,’ and ‘New Harmony’ that show an acceptable degree of field tolerance to DED^7, 8^. Identification of defense mechanisms in these tolerant cultivars could lead to the development of novel elm genotypes with robust defense lines against DED, either through classical breeding approaches (e.g. hybridization) or through genetic modification strategies.

A recent study aimed at investigating tolerance and susceptibility to DED has indicated that the different phenotypic responses of elm genotypes to DED might be attributable to differences in the timing and magnitude of defense-related transcripts and molecules (e.g. plant hormones) after infection^15^. However, it was not clear from this study whether xylem tissues, in which the infection takes place, contribute to such molecular and biochemical differences between genotypes. Reports on xylem responses to vascular wilt disease are mostly based on the proteomic analyses of xylem sap^9–12^ after different scenarios. For instance, the comparisons between xylem sap proteome of tomato plants infected with the xylem-colonizing fungus, *Fusarium oxysporium* versus healthy plants show many defense-related proteins, including pathogenesis-related proteins (PR1, PR2, PR3, PR5) and peroxidases that are specific for infection^11^. Similarly, the analysis of the xylem sap of different grape species indicates that species tolerant to the vascular wilt caused by Pierce’s disease (PD) accumulate more defense-related proteins (i.e. beta-1–3-glucanase and peroxidase) than susceptible species^9^. Nevertheless, the same set of proteins have also been identified in the xylem sap of healthy plants of *Brassica napus* ^13^ and maize^14^, suggesting that absence or presence of these proteins in the xylem is not well-attributed to the tolerance status of genotypes. Instead, it might be the relative expression and induction kinetics of these proteins after infection that define the compatible and incompatible host-pathogen interactions. Furthermore, the analysis of xylem sap could predict, but not exclusively represent, the local defense pathways in the infected xylem elements. This is because the xylem-phloem translocation loops could contribute to the accumulation of these proteins away from their origin of synthesis and hence the proteins identified in the xylem sap might not be necessarily *de-novo* synthesized by xylem elements. For these reasons, in the present study, the quantification of defense-related transcripts and hormones was performed on bark and wood tissues, manually separated from elm trees after different times of fungal infection, aiming to explore how xylem tissues would specifically contribute to the defense against DED.

The comparisons between tolerant and susceptible American elm clones have also shown that JA levels surge in the tolerant variety at 96 hours post-inoculation (hpi), the same time-point where most defense-related genes are triggered by the fungal infection, suggesting that JA may trigger the expression of these genes^15^. This has been demonstrated further, as methyl jasmonate (MeJA) application to elm saplings led to the induction of these genes and the expression level was dose-dependent^15^. However, it is still unclear whether xylem contributes to the biosynthesis and induction of JA in response to fungal infection. The current model of JA biosynthesis suggests that most of the key enzymatic reactions leading to JA take place in chloroplasts and peroxisomes^16^; both of which exist predominantly in leaf mesophyll cells, and are less abundant in non-vegetative tissues (i.e. xylem). Furthermore, the axial and radial translocation of JA through the plant vascular elements (xylem and phloem) has been proven^17, 18^. Together this suggests that JA detected in elm stems could have been translocated from adjacent organs, rather than being biosynthesized de novo in elm xylem elements after infection. On the other hand, recent research on lipoxygenase encoding genes, particularly linoleate 13S-lipoxygenase (13-LOX) which mediates the first step of JA-biosynthesis, shows localization of this gene in *Arabidopsis* xylem contact cells^19^, suggesting that JA biosynthesis might also take place in xylem. To examine these possibilities, the endogenous levels of three jasmonates (12-oxo phytodienoic acid (OPDA), jasmonic acid (JA) and JA- isoleucine (JA-Ile); and the transcription profiles of six JA-biosynthesis genes have been monitored in inoculated and non-inoculated bark and core tissues of two elm varieties. The levels of SA and ABA have also been assessed in this study in order to examine their roles in defense against DED and to monitor their accumulation pattern in phloem and xylem compared to JA, which despite sharing similar physio-chemical properties with these plant hormones, may display a different distribution pattern in vascular tissues^20^. Overall, the present study aimed to explore the nature and magnitude of defense responses in the xylem and phloem of elm stems after fungal infection.

## Results

### Expression of fungal genes in tolerant elm genotypes

**Figure 1:**
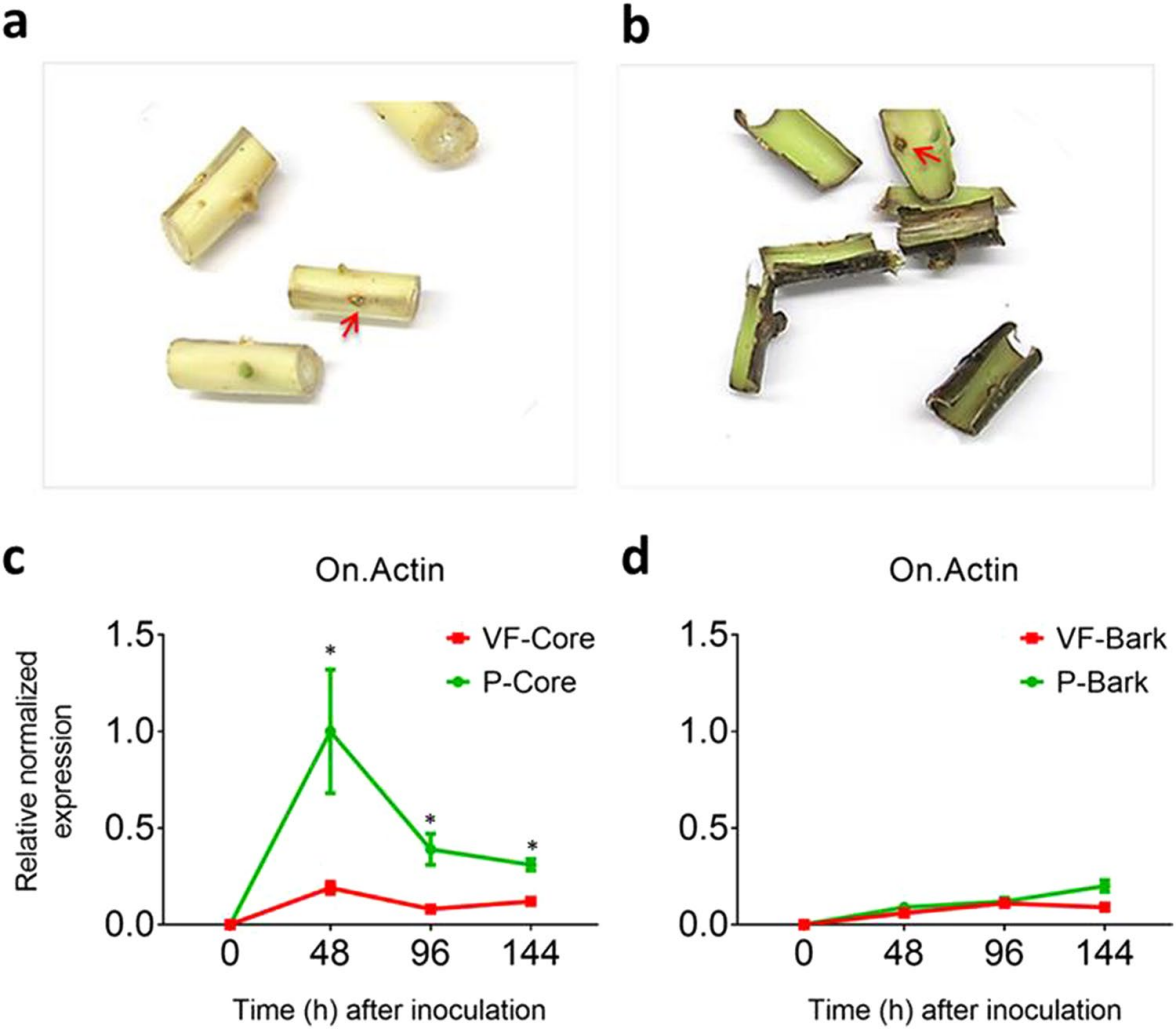
Level of fungal actin transcripts in the inoculated tissues of ‘Valley Forge’ and ‘Princeton’. (**a**,**b**) Photographs of the core and bark, respectively, of American elm stem sections inoculated with *O*. *novo-ulmi* (MH75-4O). Arrows refer to the inoculation point. (**c-d**) Expression of the fungal actin gene (*On-actin*) in mock-inoculated (0 hpi) and inoculated core and bark tissues, respectively, of ‘Valley Forge’ (VF) and ‘Princeton’ (P). The Y-axis shows the expression of *On-actin* gene normalized to the expression of three elm reference genes (*EIF 5a*, *vacuolar ATP synthase and splicing factor 3B*) and calculated relative to control (P-core at 48 hpi). Values marked with an asterisk (*) are significantly greater (≥2 times) than the corresponding time point in the other genotype (*P* < 0.05).

‘Valley Forge’ and ‘Princeton’ are two American elm varieties that show great tolerance to Dutch elm disease. The field evaluation of these varieties by the USDA indicates that ‘Valley Forge’ is more tolerant than ‘Princeton’^21^. To examine this further using the molecular detection approach described previously by Sherif *et al*.^15^, the expression of fungal actin was monitored in the bark and core tissues of both varieties within five days of inoculation with the aggressive strain (MH 75-4 O) of *O*. *novo-ulmi*. The separation of bark tissues from the core was done manually and only the area surrounding the inoculation point (nearly 2 cm^2^) was used for the molecular and biochemical analyses (Fig. 1a,b, Fig. S1). The analysis of the fungal actin (*On-Actin*) transcripts in mock-inoculated (0 hpi) and inoculated tissues (48–144 hpi) showed higher expression in ‘Princeton’ compared to ‘Valley Forge’. However, the results also indicated that the level of fungal actin transcripts decreases significantly in both varieties at 144 hpi compared to 48 hpi (Fig. 1c). Not surprisingly, the expression of *On-Actin* was significantly lower in bark compared to core tissues in both varieties, particularly at 48 hpi (Fig. 1d).

### Expression profiles of disease-responsive genes in core and bark tissues of tolerant elm varieties

**Figure 2:**
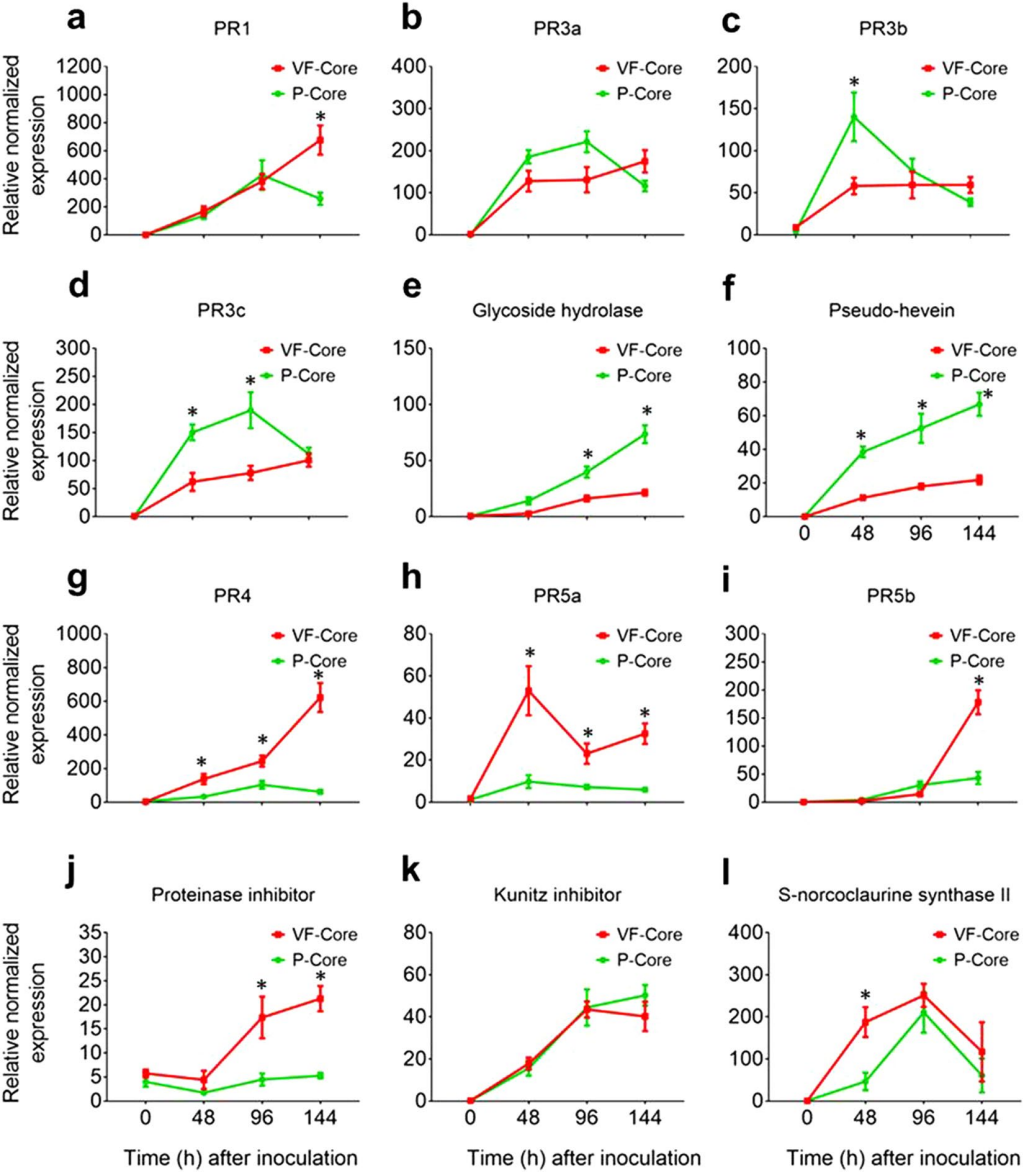
Expression of disease-responsive genes in the inoculated core tissues of ‘Valley Forge’ and ‘Princeton’. (**a**–**l**) Expression of different classes of disease responsive genes was investigated in the mock-inoculated (0 hpi) and inoculated core tissues of ‘Valley Forge’ (VF) and ‘Princeton’ (P). The expression of each gene was normalized to that of three elm reference genes (*EIF 5a*, *vacuolar ATP synthase and splicing factor 3B*) and was calculated relative to the control sample (P-bark at 0 hpi). The results are the mean ± SE of three biological replicates. Values marked with an asterisk (*) are significantly greater (≥2 times) than the control sample and the corresponding time point in the other genotype (*P* < 0.05).

**Figure 3:**
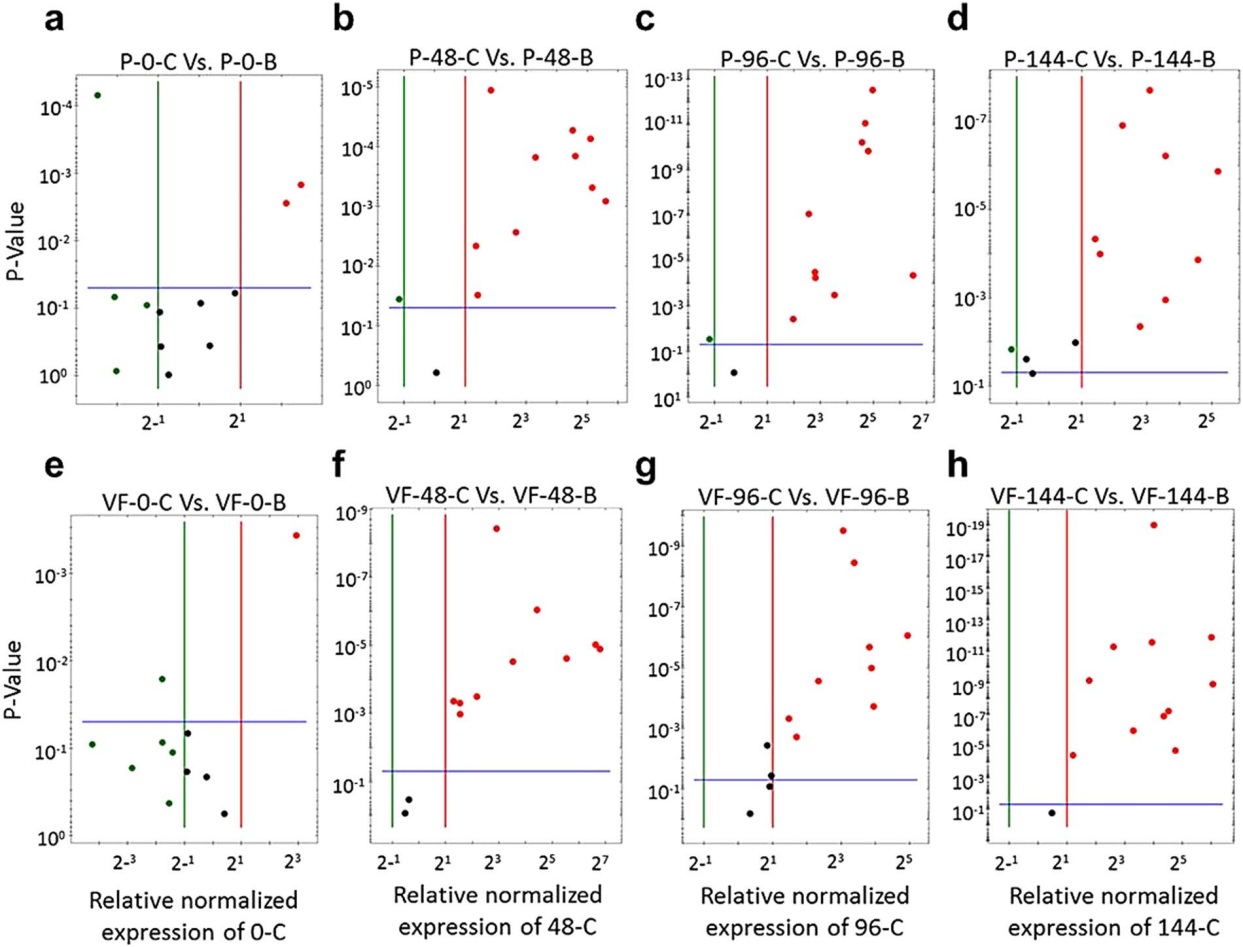
Differential expression of disease-responsive genes in the inoculated stem tissues of ‘Valley Forge’ and ‘Princeton’. (**a**–**h**) Volcano plots showing the clusters of genes expressed differentially in bark vs. core tissues of ‘Princeton’ (upper panels) and ‘Valley Forge’ (lower panels) at different time points post-inoculation. The horizontal line marks the threshold (*P* < 0.05) for defining a gene as up-regulated in bark (green) or core (red), with a combined change >2-fold.

The induction kinetics of 15 genes collectively referred to as “disease responsive genes” has previously been investigated in the stems of ‘Valley Forge’ and susceptible American elm saplings^15^. Although most of these genes have shown significant induction in response to fungal infection in tolerant and susceptible saplings compared to the control (0 hpi), the tolerance mechanism has been characterized by faster and/or higher induction of gene expression after fungal infection. In the present study, 12 of these genes were investigated in core and bark tissues of ‘Valley Forge’ and ‘Princeton’ at different time points post-inoculation. The results of this experiment can be summarized in six points. a) In the core tissues of both tolerant varieties, all genes were significantly induced (≥2-fold; *P* < 0.05) in at least one time point post-inoculation compared to the control. The only exception was the *proteinase inhibitor*, which did not show any differential induction in the inoculated core tissues of ‘Princeton’ compared to control (Fig. 2a–l). b) Genes that encode chitinases (i.e. *PR3b*, *PR3c*, *glycoside hydrolase*) or show chitinase activity (i.e. *pseudo-hevein*) were significantly higher (≥2-fold; *P* < 0.05) in the inoculated core tissues of ‘Princeton’ compared to ‘Valley Forge’ (Fig. 2c,d,e and f); whereas genes encode PR1, PR4, PR5a, PR5b, proteinase inhibitor and S-norcoclaurine synthase II were significantly higher in ‘Valley Forge’ (Fig. 2a,g,h,i,j,l). c) Most genes reached their maximum expression levels at 96 or 144 hpi in the inoculated core tissues of both varieties. d) Most genes showed significantly higher expression (≥2-fold; *P* < 0.05) in the core compared to bark tissues after all time points post-inoculation (Fig. 3a–h). The relative expression values of some genes were up to 2^7^-fold higher in the core compared to bark tissues after fungal infection. e) Despite showing significantly less expression in the bark, most disease-responsive genes exhibited similar expression profiles to those in the core tissues of both varieties. For instance, transcripts of *PR1*, *PR4*, *PR5a* and *S-norcoclaurine synthase II* were significantly more abundant in the inoculated bark tissues of ‘Valley Forge’ than ‘Princeton; whereas genes encode chitinases or showing chitinase activity were more expressed in ‘Princeton’ (Fig. S2). f) Most disease responsive genes showed no significant induction in the mock-inoculated (0 hpi) core and bark tissues of both varieties (Fig. 3a,e).

### The endogenous levels of jasmonates, SA and ABA in ‘Valley Forge’ and ‘Princeton’

**Figure 4:**
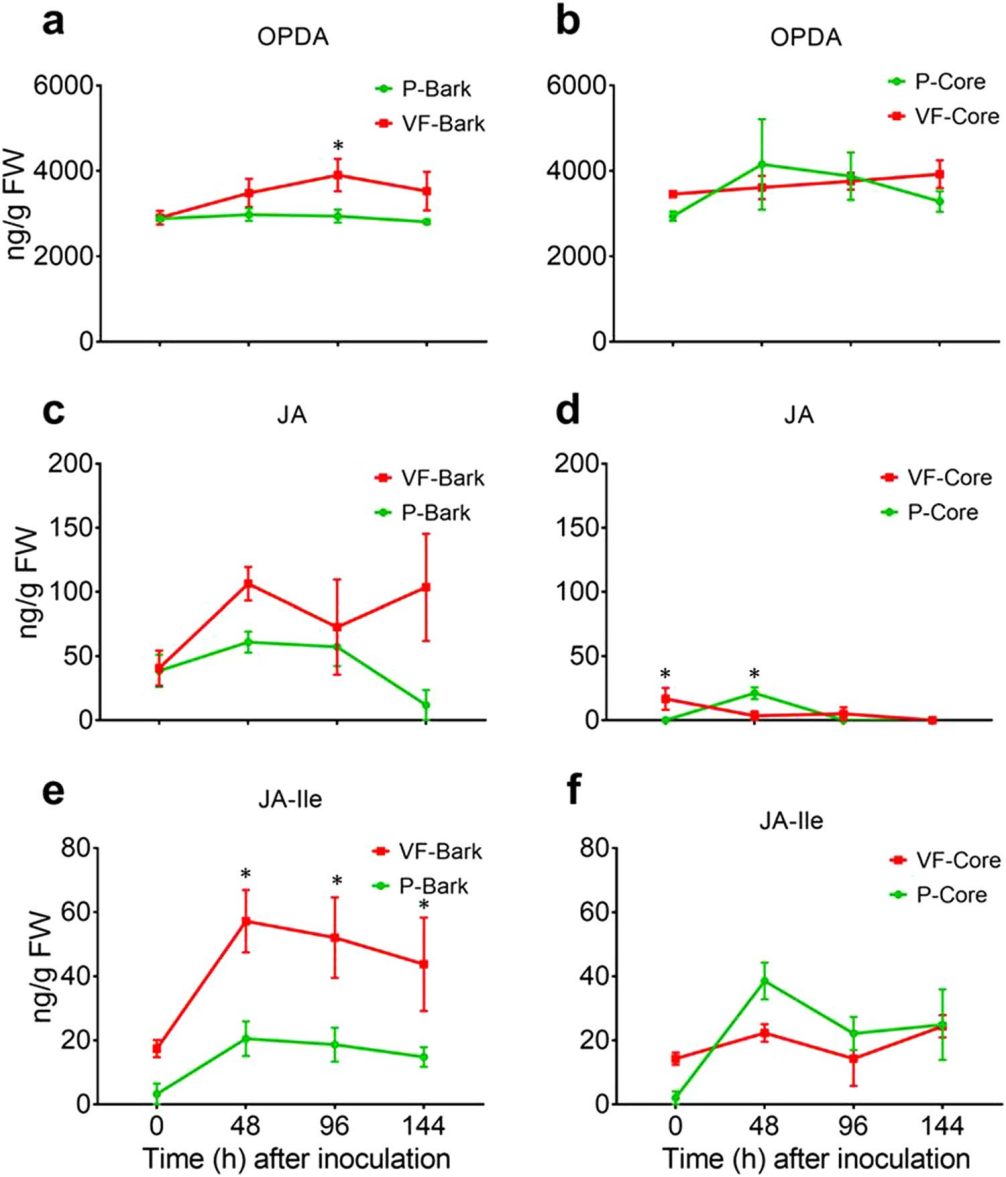
Levels of jasmonates in the inoculated stem tissues of ‘Valley Forge’ and ‘Princeton’. (**a–f**) The level of 12-oxo phytodienoic acid (OPDA), jasmonic acid (JA) and JA- isoleucine (JA-Ile), respectively, in the bark (**a**,**c**,**e**) and core (**b**,**d**,**f**) tissues of ‘Valley Forge’ (VF) and ‘Princeton’ (P) at different time points of inoculation with the *O*. *novo-ulmi* (MH75-4O). The results are the mean ± SE of three biological replicates. Values marked with an asterisk (*) are significantly higher than the control sample (P-bark at 0 hpi) and the corresponding time point in the other genotype (*P* < 0.05).

Our previous report about tolerance of American elm to Dutch elm disease has suggested that JA might play a key role in tolerance to Dutch elm disease^15^. To examine this hypothesis further, the levels of three jasmonates (OPDA, JA and JA-Ile) were quantified in the mock-inoculated and inoculated core and bark tissues of ‘Valley Forge’ and ‘Princeton’. In general, the results indicate that the levels of OPDA, JA and JA-Ile in the inoculated bark tissues of ‘Valley Forge’, but not ‘Princeton’, were significantly higher compared to the mock-inoculated tissues in at least one time point post-inoculation. However, only OPDA and JA-Ile showed significant differences (*P* < 0.05) between ‘Valley Forge’ and ‘Princeton’ in the inoculated bark tissues; with JA-Ile exhibiting significantly higher levels in ‘Valley Forge’ at all time points post-inoculation (Fig. 4a,c,e). In the core tissues, however, OPDA levels did not show any change after fungal infection in both varieties, but were generally high (2940–4152 ng/g FW) and comparable to those detected in the bark (2550–3904 ng/g FW). The levels of JA and JA-Ile were significantly induced in the inoculated core tissues of ‘Princeton’, but not ‘Valley Forge’, in at least one time-point post-inoculation. However, no significant differences in JA-Ile levels were detected between varieties, probably due to the high basal levels (14.2 ng/g FW) of this compound in the core of ‘Valley Forge’ compared to ‘Princeton’ (2.04 ng/g FW). The levels of JA were generally lower in the core tissues (0–21.2 ng/g FW) of both varieties compared to those detected in the bark tissues (12–106 ng/g FW) (Fig. 4b,d,f).

**Figure 5:**
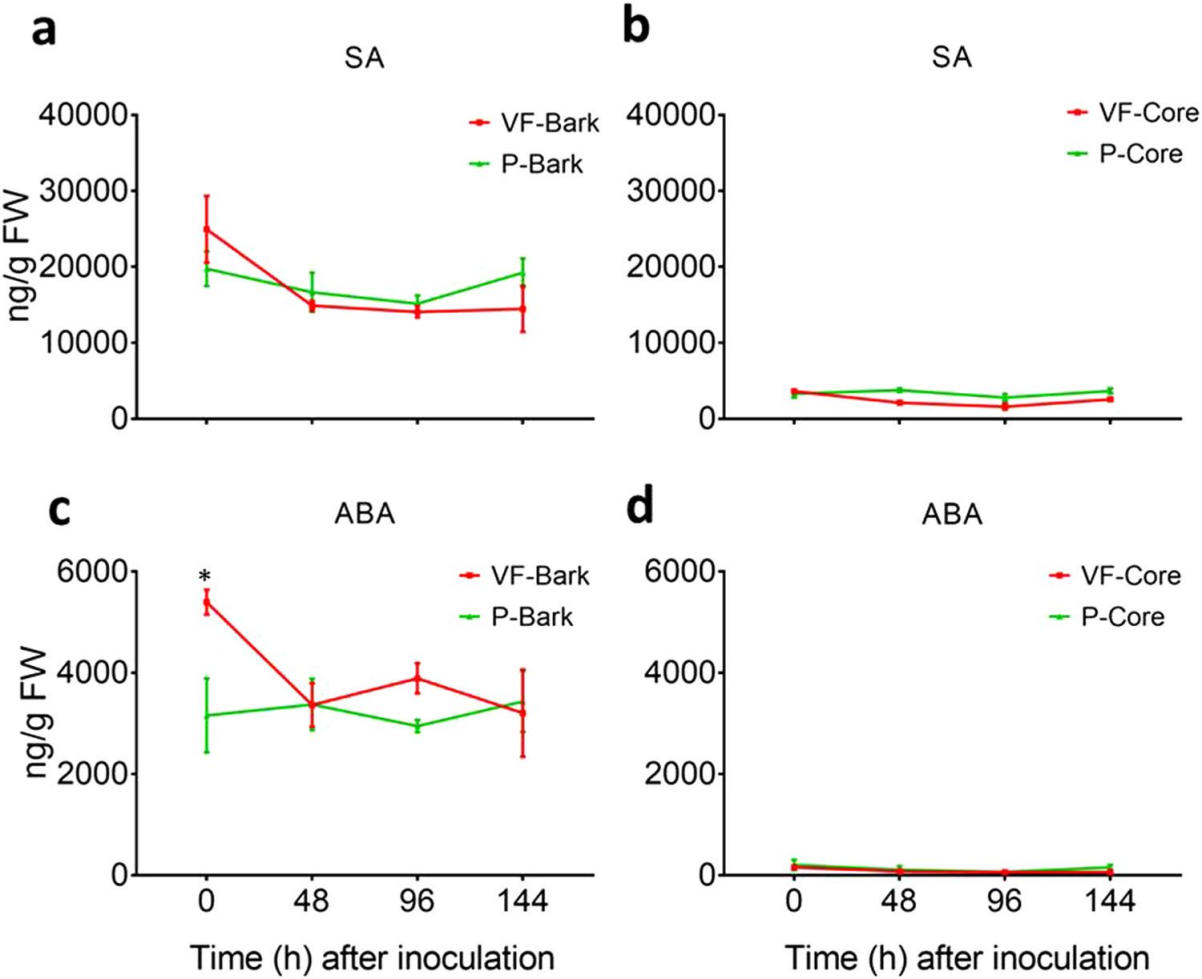
Levels of salicylic acid (SA) and abscisic acid (ABA) in the inoculated stem tissues of ‘Valley Forge’ and ‘Princeton’. (**a**,**b**) The level of SA (ng/g FW) in the bark and core tissues, respectively, of ‘Valley Forge’ (VF) and ‘Princeton’ (P) at 0, 48, 96 and 144 hpi. (**c**,**d**) the level of ABA (ng/g FW) in the bark and core tissues, respectively, of ‘Valley Forge’ (VF) and ‘Princeton’ (P) at different time points of inoculation with the fungus. The results are the mean ± SE of three biological replicates. Values marked with an asterisk (*) are significantly higher than the control sample (P-bark at 0 hpi) and the corresponding time point in the other genotype (*P* < 0.05).

Other plant hormones that have often been correlated to defense against invading pathogens are SA and ABA. However, in the present study, the levels of SA showed no significant change in the core and bark tissues of ‘Valley Forge’ and ‘Princeton’ at any time point prior or post-inoculation. However, it was noted that SA levels were significantly higher (*P* < 0.05) in the bark (14075–24952 ng/g FW) compared to core (1591–3776 ng/g FW) tissues of both varieties (Fig. 5a,b). Similarly, ABA levels did not show any significant change in the bark and core tissues of both varieties at any time point post-inoculation. However, the basal levels of ABA in the mock-inoculated bark tissues (0 hpi) of ‘Valley Forge’ were significantly higher (*P* < 0.05) compared to those in ‘Princeton’. Similar to SA, the levels of ABA in the bark (2947–5396 ng/g FW) were many folds higher than those in the core tissues (62–204 ng/g FW) of both varieties (Fig. 5c,d).

### Expression profiles of JA-biosynthesis genes in the American elm stem tissues

**Figure 6:**
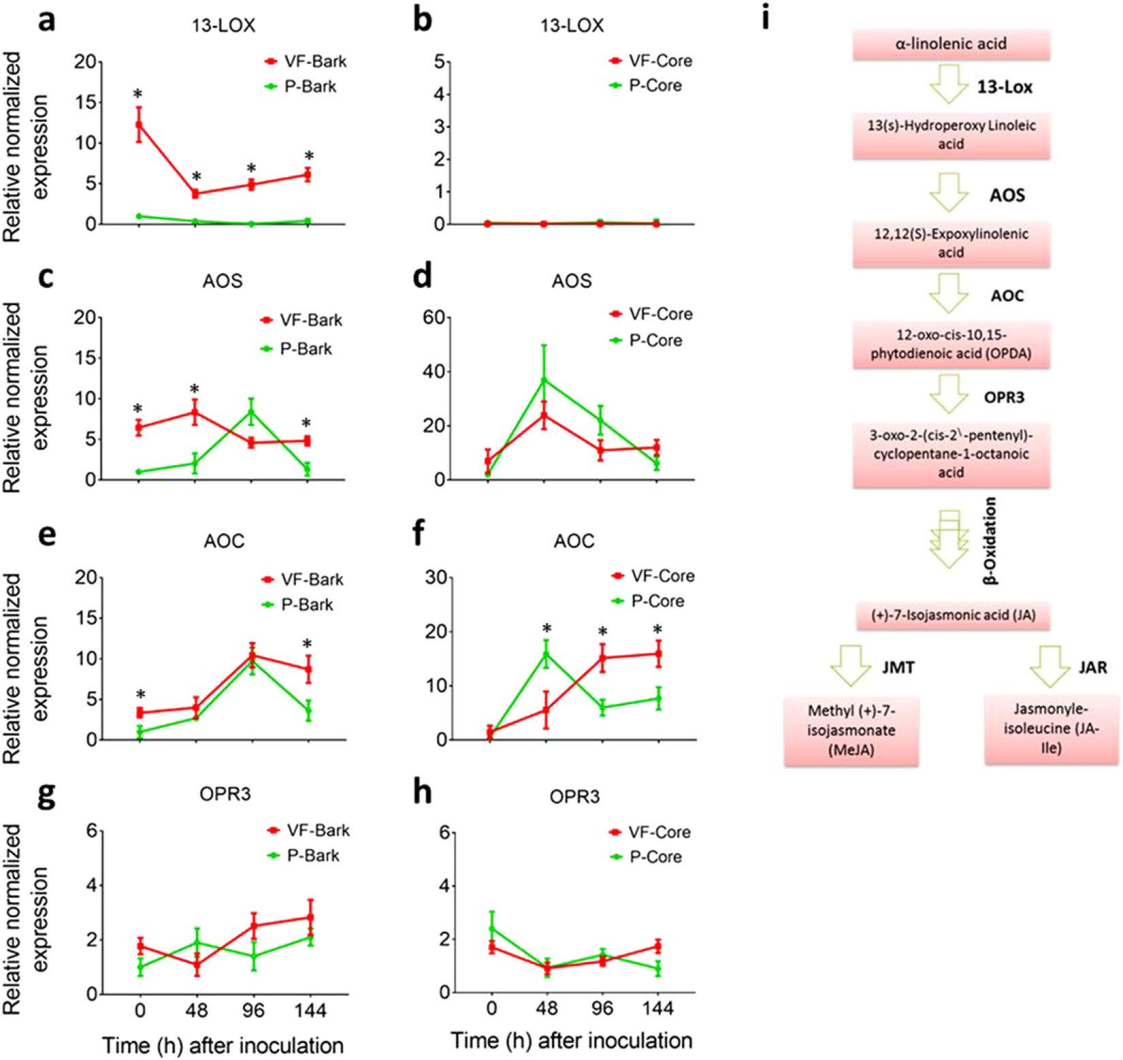
Expression of JA-biosynthesis genes in infected and non-infected tissues of ‘Valley Forge’ and ‘Princeton’. (**a–h**) The relative normalized expression of genes encode linoleate 13S-lipoxygenase (13S-LOX), allene oxide synthase (AOS), allene oxide cyclase (AOC) and OPDA-reductase 3 (OPR3), respectively, in the mock-inoculated (0 hpi) and inoculated bark (**a**,**c**,**e**,**g**) and core (**b**,**d**,**f**,**h**) tissues of Valley Forge’ (VF) and ‘Princeton’ (P). The expression of each gene was normalized to that of three elm reference genes (*EIF 5a*, *vacuolar ATP synthase and splicing factor 3B*) and was calculated relative to the control sample (P-bark at 0 hpi). The results are the mean ± SE of three biological replicates. Values marked with an asterisk (*) are significantly greater (≥2 times) than the control sample and the corresponding time point in the other genotype (P < 0.05). (**i**) A schematic overview of the major steps and enzymes in the JA biosynthesis pathway.

**Figure 7:**
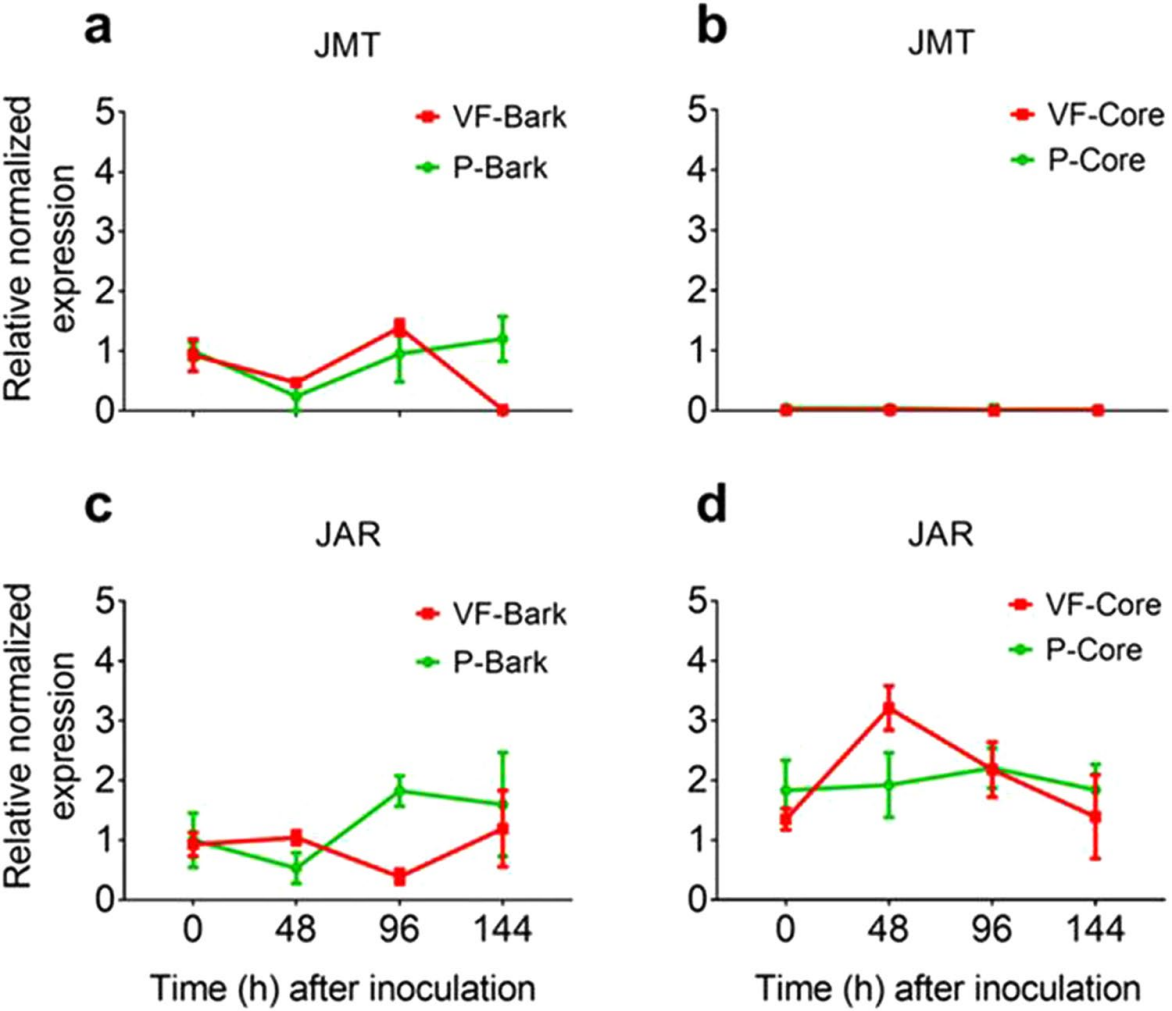
Expression of JA-methyltransferase (JMT) and JA-resistance (JAR) genes in inoculated elm stems. (**a–d**) the relative normalized expression of *JMT* and *JAR* in bark (**a**,**c**) and core (**b**,**d**) tissues, respectively, of Valley Forge’ (VF) and ‘Princeton’ (P). The expression of each gene was normalized to that of three elm reference genes (*EIF 5a*, *vacuolar ATP synthase and splicing factor 3B*) and was calculated relative to the control sample (P-bark at 0 hpi). The results are the mean ± SE of three biological replicates.

Six genes encoding 13S-LOX, allene oxide synthase (AOS), allene oxide cyclase (AOC), OPDA-reductase 3 (OPR3), JA-methyltransferase (JMT) and JA-resistance (JAR) were isolated from a cDNA library prepared from the infected bark tissues of ‘Valley Forge’. The partial sequences of these genes were deposited in Gene Bank (Accession numbers, KY866673-78) and were used further to design qRT-PCR primers in order to monitor the expression of these genes in different tissues and after different conditions (Fig. 6a–h). The results of this experiment can be concluded in four main points: (a) Transcripts of the five JA-biosynthesis genes were detected in all the cDNAs generated from the bark and core tissues of ‘Valley Forge’ and ‘Princeton’, suggesting that enzyme products of these genes also exist in these tissues. (b) Genes that encode enzymes upstream OPDA (i.e. *13S-LOX*, *AOS* and *AOC*) showed significantly higher transcript levels in the inoculated bark tissue of ‘Valley Forge’ in at least one time point post-inoculation and also showed higher basal levels (0 hpi) in the bark tissues of ‘Valley Forge’ compared to ‘Princeton’ (Fig. 6a,c,e). (c) In core tissues, the expression of *13S-LOX* was significantly lower compared to that in bark tissues under all conditions; whereas the relative normalized expression of *AOS* and *AOC* was generally higher in the core than in bark tissues (Fig. 6b,d,f). Similar to those in bark, transcripts of *AOS* and *AOC* showed significant induction in response to fungal infection in the inoculated core tissues of both varieties in at least one time-point post-inoculation (Fig. 6d,f). (d) Genes encoding enzymes downstream of OPDA (i.e. *OPR3*, *JMT* and *JAR*) did not show any significant induction in response to fungal infection in the core and bark tissues of both varieties (Figs 6g,h and 7a–d).

## Discussion

Several studies on pathosystems involving annual plants and vascular wilt pathogens conclude that resistance to vascular wilt diseases is tissue specific. For instance, transcriptome changes in tomato plants infected by a fungal foliar pathogen (i.e. *Cladosporium fulvum*) were different and in some cases opposite to those noticed after the infection of plants with a vascular wilt fungal pathogen (i.e. *Verticillium dahlia*)^22^. In another example involving the *Arabidopsis WAT1* mutant, which is implicated in resistance to vascular wilt, but not foliar pathogens; the plants showed resistance only when the pathogens were directly delivered into the vascular system, but not when injected into the leaf mesophyll tissues^23^. Although these studies and others^24, 25^ suggest the specific nature of xylem-mediated defense responses, the physical separation of xylem from surrounding tissues was thought to provide unambiguous evidence about the role the xylem plays during interaction with vascular wilt pathogens.

The findings of the present study indicate through many lines of evidence that the xylem not only contribute to the synthesis but also to the delivery of defense signals against DED. First, unlike other macromolecules (e.g. proteins, small peptides) that exist in the xylem sap and be translocated through the phloem-xylem translocation loop, xylem sap is RNA-free^26^. Hence, the existence of defense-related transcripts in elm wood tissues and their accumulation after fungal infection postulates local transcriptional activity of xylem cells surrounding the infection site. Second, the induction of specific classes of genes in ‘Valley Forge’ and ‘Princeton’ suggests different perceptions of fungal-driven signals (e.g. pathogen-associated molecular patterns (PAMPs), fungal effectors) by these varieties, which principally necessitates localized interactions between ligand/receptors counterparts from fungus and xylem cells, respectively. Third, the similar expression profiles of defense-related genes between wood and bark tissues (Fig. 2 and Fig. S1) suggests the delivery of the defense signal through xylem flow, not phloem flow, as the latter would only deliver a generic wound signal that would not lead to such specific induction of defense-related genes in both elm varieties.

The accumulation of the three jasmonates (OPDA, JA and JA-Ile) in the inoculated bark and core tissues of ‘Valley Forge’ and ‘Princeton’ after fungal infection indicates that tolerance mechanisms in these varieties are, at least partially, controlled by JA signaling pathway. However, the accumulation of jasmonates in the bark and core tissues after fungal infection does not necessarily reflect their biosynthesis in the phloem and xylem, respectively. Indeed, the higher accumulation of JA, SA and ABA in the bark than in core tissues could be due to the ion trap mechanism, which predicts that the protonated forms of these weak organic acids can permeate membranes and migrate toward sieve elements (SEs) under the influence of the alkaline/neutral environment of the phloem and the relatively weak acidic nature of the xylem^20, 32, 33^. Nevertheless, the detection and the transcriptional induction of three JA-biosynthesis genes (*13S-LOX*, *AOS* and/or *AOC*) in the bark and core tissues of ‘Valley Forge’ and ‘Princeton’ suggest that de novo biosynthesis of JA in response to fungal infection could take place in these tissues. Indeed, the enzymes involved in JA-biosynthesis have previously been detected in the SEs and companion cells of tomato plants^34, 35^, and in phloem exudates of other plant species^36, 37^. Moreover, two of the four 13-LOX genes in *Arabidopsis*, *LOX4* and *LOX6*, have shown localization in phloem-associated cells and xylem contact cells, respectively^19, 38^. Although the core tissues of ‘Valley Forge’ and ‘Princeton’ did not show any induction of *13-LOX* transcripts post inoculation (Fig. 6), it is possible that other forms of 13-LOX enzymes in American elm are involved in JA biosynthesis in the xylem. Although the lateral exchange of jasmonates between the xylem and phloem in response to pathogen signals (i.e. flg22) has been previously hypothesized^20^, the accumulation pattern of JA-related metabolites or transcripts in bark and core tissues does not support this hypothesis.

JA biosynthesis and/or signaling pathways are not always associated with defense against vascular wilt pathogens. Indeed, tolerance to major vascular wilt fungi, i.e. *F*. *oxysporum* and *V*. *longisporum* is enhanced in JA-insensitive mutants (e.g. *myc2*, *coi1*, *pft1*)^27–29^. On the other hand, the application of SA leads to more resistance against these pathogens. In the present study, the levels of SA did not show major changes in bark and core tissues of both tolerant elm varieties after infection. However, in a previous study, SA (2 mM) has increased the tolerance to DED when applied 24 hpi to 4-year-old American elm saplings; whereas the sole application of MeJA (100 µM) led to the same level of susceptibility as control^15^. It has also been shown that the application of MeJA is not effective in enhancing the tolerance of *Ulmus minor* against *O*. *novo-ulmi* ^30^. These data reveal that *O*. *novo-ulmi* fungus is a hemibiotrophic pathogen, with an initial biotrophic phase and a later necrotrophic phases. Since it has become evident, with some exceptions, that SA provides resistance to biotrophs, whereas JA providence resistance to necrotrophs^31^; the induction of SA at early stages (probably before 48 hpi) and the later induction of JA might be linked to tolerance to DED. This also explains the significant effect of the combined treatment of SA and MeJA in reducing fungal growth in elm saplings when applied 24 and 96 hpi, respectively^15^.

The expression of certain classes of pathogenesis-related (PR) proteins in the infected tissues of ‘Valley Forge’ and ‘Princeton’ implies different defense mechanisms in these varieties. In brief, the degradation of fungal cell walls through PR2 (glycoside hydrolases) and PR3 (chitinases) group of proteins represents the primary defense strategy in ‘Princeton’. Although these proteins hydrolyze chitin in fungal cell walls and show high anti-fungal activity, some fungal species have evolved mechanisms to overcome this defense barrier. Among these mechanisms is the secretion of the LysM effector proteins that suppress chitin-triggered immunity, and the secretion of proteolytic enzymes that target and cleave host chitinases^39–41^. Both of these mechanisms have been recruited by vascular wilt fungi and could also be used by *O*. *novo-ulmi*.

On the other hand, the defense response of ‘Valley Forge’ is characterized by the induction of three classes of PR proteins (PR4, PR5 and PR6). Interestingly, PR4 proteins have been considered among the markers for JA-dependent defense pathways against necrotrophic pathogens^42^, which agrees with the high JA levels observed in ‘Valley Forge’ and the proposed necrotrophic phase of ‘*O*. *novo-ulmi*’. These proteins show *in-vitro* anti-fungal activity against a number of pathogenic fungi, confer resistance when overexpressed in transgenic plants, and function as a positive mediator of plant cell death^43–46^. Thaumatin-like proteins (PR5) also show anti-fungal activity *in-vitro* ^47, 48^ and are thought to mediate this role through their induction of programmed cell death in fungi, inhibition of fungal enzymes, and/or by rupturing fungal membranes^49–52^. Similar to PR4, proteinase inhibitors (PIs) (PR6) proteins are often associated with JA-mediated defense responses against necrotrophs and herbivores^53, 54^. Pathogenic fungi secrete proteases to suppress host defenses and to help in colonization^55^. Therefore, the induction of PIs in ‘Valley Forge’ after fungal infection represents another effective line of defense against *O*. *novo-ulmi*. In general, the defense responses in ‘Valley Forge’ is not only different from that of ‘Princeton’, but they are diverse and more effective in impeding fungal growth and development inside the colonized xylem vessels, as demonstrated by the decreased expression of fungal ‘*On-Actin*’ in the inoculated stems of ‘Valley Forge’ compared to ‘Princeton’ (Fig. 1c,d).

The analysis of transcriptional and hormonal changes in the detached core and bark tissues provided a reliable experimental model to help understand the distinct roles of xylem and phloem during incompatible interactions with vascular wilt fungi such as *Ophiostoma* spp. The present findings indicated that defense tactics are not uniform among tolerant elm varieties and revealed that de novo biosynthesis of jasmonates occurs in the xylem and phloem tissues during local immune responses against DED.

## Materials and Methods

### Plant materials

Five-year-old American elm ‘Valley Forge’ and ‘Princeton’ saplings were purchased from a commercial nursery (NVK Connon Nurseries, Dundas, ON, Canada) and maintained in an open field at University of Guelph for two weeks prior to fungal infection.

### Inoculations of American elm saplings

The preparation of *O*. *novo-ulmi* (MH75- 4 O) inoculum and the inoculation processes were performed according to the methods described previously^15^. Stem plugs (~2 cm^2^) collected from the mock-inoculated tissues (0 hpi) and inoculated tissues (48–144 hpi) were flash-frozen in liquid nitrogen and stored at −80 °C until further analyses. The experiment was performed in triplicate with each sapling representing one replicate. The inoculations of each sapling were done at twig crotches only and two sets of stem plugs were collected from each sapling; one set for gene expression analysis and another set for the hormone quantification analysis.

### RNA extraction and quantitative gene expression

The extraction of RNA from bark and core tissues was done according to the modified CTAB method described previously^15^.The only modification of this method was the separation of bark from core tissues before tissue grinding. To perform this step, frozen stem plugs were obtained from −80 °C and kept on ice for 15 min to facilitate the manual separation of the bark from core tissues. The separated tissues were then frozen in liquid nitrogen again.

The preparation of cDNA libraries, primers used for qRT-PCR, analysis of gene expression, statistical analysis of the results and representation of the relative normalized expression values in volcano plots were done according to the method described previously^15^.

### Isolation of the JA-biosynthesis genes from American elm

The partial sequences of *13S-LOX*, *AOS*, *OAC*, *OPR3*, *JMT and JAR* were isolated from the cDNA library generated from the inoculated bark tissues of ‘Valley Forge’, using reverse transcription polymerase chain reaction (RT-PCR) and primers in Table S1. Primers to amplify these genes were designed according to the available sequences of their orthologs in the GeneBank. The resulting PCR fragments were cloned into pGEM-T easy vector (Promega, Madison, WI, USA), sequenced and compared with database sequences using the BLAST program. The sequences of these genes were deposited in the National Center for Biotechnology Information (NCBI) database. Primers to study the expression of these genes are listed in Table S2.

### Quantification of jasmonates, SA and ABA in American elm tissues

Ground samples (~250 mg) were suspended in 1 mL of extraction solvent which was comprised of 50% methanol (MS Grade, Fisher Scientific, Canada; MeOH) and 4% acetic acid (glacial, Fisher Scientific, Canada) in Milli-Q water. Samples were then sonicated for 15 min on ice and spun down (2 min, 13000 rpm) and, the supernatant removed. Supernatant was then filtered through a 0.45 µm centrifuge filter (Millipore; 1 min, 13 0000 rpm) and the flow through was diluted five times in 10 mM ammonium acetate pH 9, adjusted with ammonium hydroxide (Sigma Aldrich, Canada).

For quantification of samples by ultra performance liquid chromatography (UPLC) - mass spectrometry (MS), 5 µL of sample was injected onto a Waters Acquity BEH Column (2.1 × 50 mm, i.d. 2.1mm, 1.7 µm) on a Waters Acquity Classic UPLC system with single quadrupole MS detection (Waters QDa, Waters, Canada). Samples were run on a gradient with A −10 mM ammonium acetate pH 9, adjusted with ammonium hydroxide; B −100% MeOH with initial conditions of 95% A 5% B increased to 5% A 95% B over 4.5 min using an Empower curve of 8. Column temperature was 40 °C and flow rate was 0.5 mL/min. Compounds were monitored in positive mode in single ion recording (SIR) mode and quantified using standard curves (see Table S3 for MS parameters). In all cases, probe temperature was 500 °C with a gain of 5. Jasmonic acid, salicylic acid and abscisic acid standards were analytical grade and purchased from Sigma Aldrich, Canada, while JA-Ile and OPDA standards were purchased from Olchemim (Czech Republic).

## Electronic supplementary material

Supplementary Information
