# Supplementary material for: Bark and wood tissues of American elm exhibit distinct responses to Dutch elm disease
Source: Sci Rep. 2017 Aug 2;7:7114. doi: 10.1038/s41598-017-07779-4 (PMC5540924; doi:10.1038/s41598-017-07779-4)
Supplement: Supplementary file 1 — Supplementary Information [file 41598_2017_7779_MOESM1_ESM.pdf]

Bark and wood tissues of American elm exhibit distinct responses to Dutch elm disease

Sherif S.M.[1, 2], Erland L.A.[1], Shukla M.R.[1], Saxena P.K[1*].

[1]Gosling Research Institute for Plant Preservation, Department of Plant Agriculture, University of Guelph, Guelph, ON, Canada

[2]Virginia Tech, Alson H. Smith, Jr. Agricultural Research and Extension Center, Winchester, VA, USA

***Corresponding author**: Praveen K. Saxena

Address: Gosling Research Institute for Plant Preservation, Department of Plant Agriculture, University of Guelph, Guelph, ON, Canada, N1G 2W1

Telephone: +1 (519) 824-4120, Ext. 52495

Fax: +1 (519) 767-0755

E-mail:psaxena@uoguelph.ca

Table S1. Primers to isolate the partial sequences of JA-biosynthesis genes from elm cDNA libraries

Gene Name	Accession #	Primer Sequence
**13-LOX-F**	KY866673	GATTTTGAGAGGCAATGGAGAAGG
**13-LOX-R**		TCACACTAAGTTGCCTATTTGTTG
**AOS-F**	KY866674	CAAATCAGTGGCTTATGAAGTTCTT
**AOS-R**		TTCATATACTATATCCCTTACATT
**AOC-F**	KY866675	ATCTATACCGGAGACTTGCAAAAA
**AOC-R**		CTCCGAAGGCAAATCCCCAATTC
**OPR3-F**	KY866676	AGCACTAGCCACAAAGAAACCTCCAT
**OPR3-R**		CCCAACAAAGCGAGAGAATCTAACGTG
**JMT-F**	KY866677	TTCTTTTTACGGAAGAGTGTTCCC
**JMT-R**		CGAAATAGATCATCCATAATAACA
**JAR-F**	KY866678	CTCGTCCATTTCTTTTTCTGCAGAGAA
**JAR-R**		TGTTTCGGATCCCCGGAGAAGTAATCC

Table S2. Primers to study the expression of JA-biosynthesis genes in inoculated core and bark tissues of 'Valley Forge' and 'Princeton' after fungal infection

Gene Name	Accession #	Primer Sequence
**13-LOX-F**	KY866673	TGAGCCCCCTGAAACAATA
**13-LOX-R**		ACCACAAGGGTTGAGTCCA
**AOS-F**	KY866674	TTTGGTCAAACGGGAGAGA
**AOS-R**		CAACACTACCCTGGCCACT
**AOC-F**	KY866675	GACTTGCAAAAACGGTTGG
**AOC-R**		CCTCGTACCGGTCTCCTTT
**OPR3-F**	KY866676	TTGTGTTCGATGACCCTCT
**OPR3-R**		ACCACAAGCTGACCAAAGC
**JMT-F**	KY866677	AGCTCGAGCATTGATGACC
**JMT-R**		CTCAGGGCTTGGAGCATAA
**JAR-F**	KY866678	AACCAAAGGGGGACTTGCT
**JAR-R**		AGCATTGGGATTGGAACGC

Table S3. MS parameters for analytes run in SIR mode. ESI - electrospray ionization; m/z - mass to charge ratio

Analyte	m/z	Ionization mode	Cone voltage (V)
**SA**	137	ESI-	10
**JA**	209	ESI-	15
**JA-Ile**	322	ESI-	15
**OPDA**	291	ESI-	15
**ABA**	265	ESI+	10

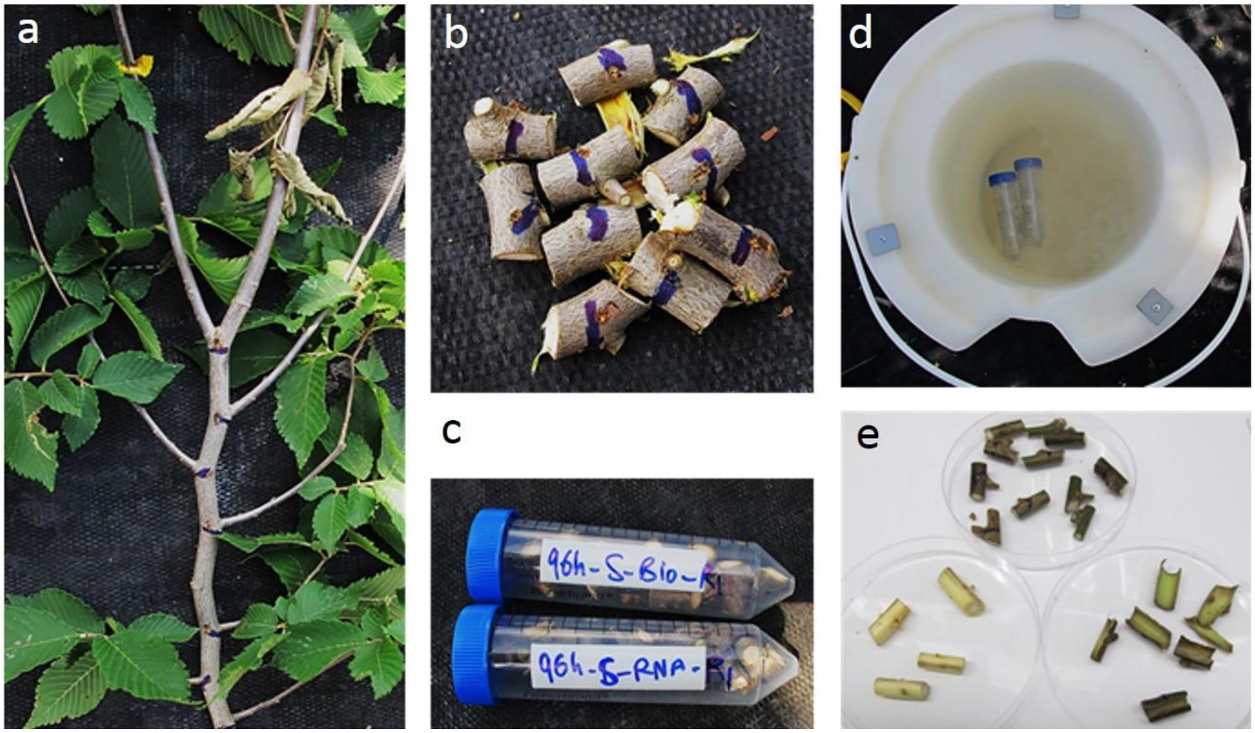

Figure S1: Collection of bark and wood tissues from inoculated elm stems for hormonal and gene expression analyses. a) The inoculation points were made inside or close to twig crotches using an electric drill**. b)** The area surrounding the inoculation point (nearly 2 cm^2) was collected using a tree pruner. **c)** Two sets of stem plugs were collected from each sapling; one set for gene expression analysis and another set for the hormone quantification analysis. **d)** Samples were flash-frozen in liquid nitrogen and stored at -80 ^{o}C until further analyses. **e)** The bark and wood tissues were manually detached, frozen again in liquid nitrogen and used directly for RNA extraction or hormone quantification.

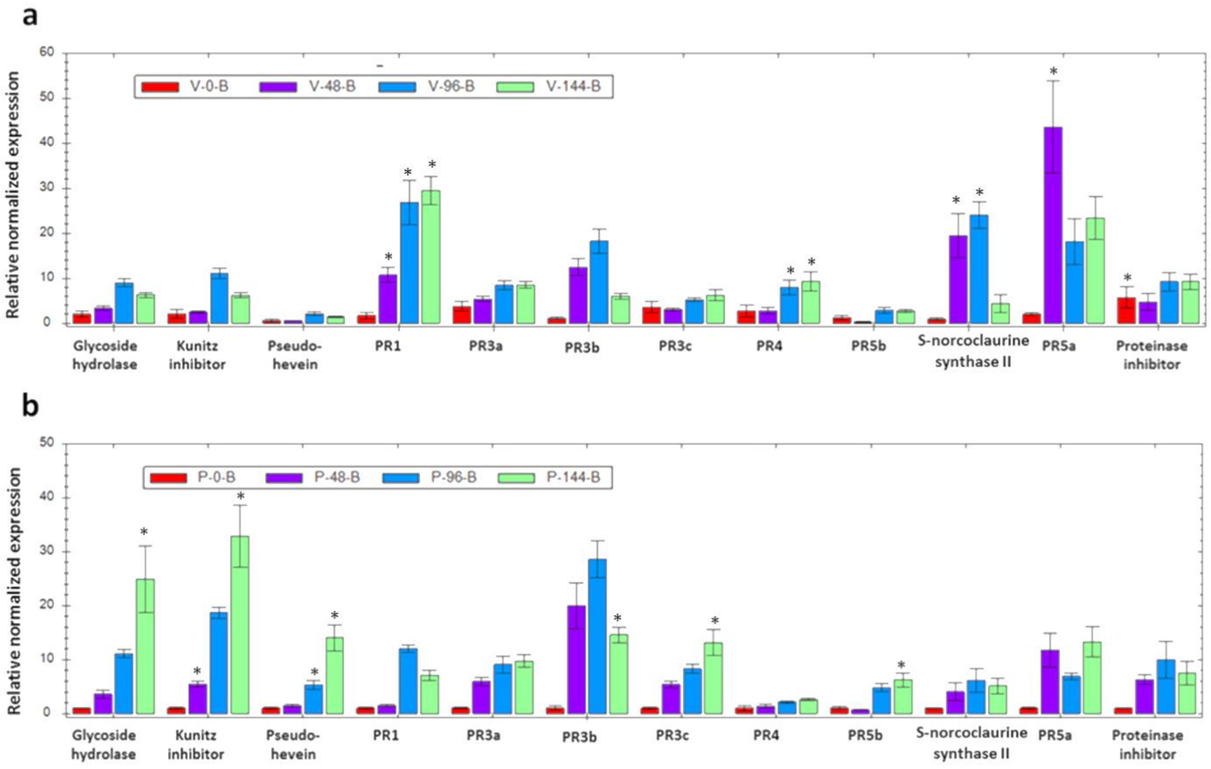

Figure S2: Expression of disease-responsive genes in the inoculated bark tissues of 'Valley Forge' and 'Princeton'. Expression of different classes of disease responsive genes was investigated in the mock-inoculated (0 hpi) and inoculated bark tissues of 'Valley Forge' (VF) **(a)** and 'Princeton' (P) **(b)**. The expression of each gene was normalized to that of three elm reference genes (*EIF 5a*, *vacuolar ATP synthase and splicing factor 3B*) and was calculated relative to the control sample (P-bark at 0 hpi). The results are the mean ± SE of three biological replicates. Values marked with an asterisk (*) are significantly greater (≥ 2 times) than the control sample and the corresponding time point in the other genotype ($P < 0.05$).
